# Residue Modification and Mass Spectrometry for the Investigation of Structural and Metalation Properties of Metallothionein and Cysteine-Rich Proteins

**DOI:** 10.3390/ijms18050913

**Published:** 2017-04-26

**Authors:** Gordon W. Irvine, Martin J. Stillman

**Affiliations:** Department of Chemistry, The University of Western Ontario, London, ON N6A 3K7, Canada; girvine@uwo.ca

**Keywords:** metallothionein structure, protein dynamics, cysteine modification, covalent labeling, ESI-MS, apo-metallothionein, metal induced protein folding

## Abstract

Structural information regarding metallothioneins (MTs) has been hard to come by due to its highly dynamic nature in the absence of metal-thiolate cluster formation and crystallization difficulties. Thus, typical spectroscopic methods for structural determination are limited in their usefulness when applied to MTs. Mass spectrometric methods have revolutionized our understanding of protein dynamics, structure, and folding. Recently, advances have been made in residue modification mass spectrometry in order to probe the hard-to-characterize structure of apo- and partially metalated MTs. By using different cysteine specific alkylation reagents, time dependent electrospray ionization mass spectrometry (ESI-MS), and step-wise “snapshot” ESI-MS, we are beginning to understand the dynamics of the conformers of apo-MT and related species. In this review we highlight recent papers that use these and similar techniques for structure elucidation and attempt to explain in a concise manner the data interpretations of these complex methods. We expect increasing resolution in our picture of the structural conformations of metal-free MTs as these techniques are more widely adopted and combined with other promising tools for structural elucidation.

## 1. Introduction

Structure is of critical importance, whether it is in the design of buildings, writing of a poem, or in the function of proteins and other macromolecules [1,2,3,4]. Structure can be thought of not only as the position of elements within the whole, but also their rigidity and other individual properties that sum together to give molecular machines and engineers amazing abilities to build skyscrapers or catalyze key reactions required for life. It is because of the inexorably linked structure/function relationship that researchers devote extraordinary efforts to determine protein structure. From the crystallization of urease [5] to modern structure-predicting software [6], many tools have been developed in order to gain a glimpse into the microscopic world of protein structure.

Over billions of years, Nature has developed an immense catalogue of macromolecular structures that support and sustain life’s essential processes. This diversity of protein structure has necessitated the development of numerous characterization techniques each able to be applied to only certain types and sub-sets of proteins. Typical characterization techniques include: X-ray diffraction from crystals, nuclear magnetic resonance (NMR) spectroscopy, electron microscopy, and small angle X-ray and neutron scattering. Due to the limitations of these techniques for molecules in solution, sophisticated mass spectrometric methods have been developed to investigate protein structure including hydrogen/deuterium (H/D) exchange, tandem mass spectrometry, ion mobility mass spectrometry (IM-MS), and residue modification coupled with electrospray ionization mass spectrometry (RM-MS).

In this review we will summarize recent advances in the lesser known method, residue modification, for the determination of structure and metal-binding properties of the flexible and ill-defined metallothionein family. The RM-MS technique is particularly suited to the study of metallothioneins because of their lack of formal secondary structural elements and abundance of specifically modifiable residues.

## 2. Metallothionein

Metallothioneins (MTs) are a family of proteins, unique in their lack of aromatic amino acids [7], small size (typically less than 10 kDa) [8], high cysteine content (~30%) [9], and flexibility that allows them to adopt many configurations, and this unique flexibility allows MTs to form metal thiolate clusters with a variety of metals [2,10]. The lack of a rigid structure allows MTs to coordinate a number of metals including essential metals like zinc and copper [11], toxic metals like cadmium [12] and mercury [13], and exotic metals like uranium [14] and technetium [15]. This flexibility, while fundamental to their function, makes MTs extremely difficult to characterize structurally.

MTs exist in almost all forms of life from bacteria to more complex invertebrates and mammals but are curiously absent from the genomes of Cnidarians [16,17,18,19]. While structural diversity exists for the many MTs in these divergent kingdoms of life, most consist of two separate metal binding domains that form a “dumbbell” shape when in their holo-form [20]. While other recent reviews have summarized advances in the characterization of plant, bacterial, and other non-mammalian MTs [2,21,22], we will focus on mammalian MTs.

**Figure 1:**
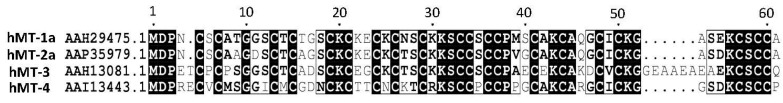
Clustal Omega sequence alignment of the major human metallothionein isoforms with conserved amino acids highlighted in black. Generated with ESPript 3.0.

Mammalian MTs have four main isoforms: MT-1 and MT-2 expressed constitutively with a higher content in liver and kidneys [23,24], MT-3 expressed mainly in the brain and central nervous system [25], and MT-4 expressed in cornified, stratified, squamous epithelium, a protective tissue found on many organs including the skin, tongue, and vagina [26]. The conserved sequences between the mammalian isoforms contain the 20 cysteines that are essential for metal binding, Figure 1.

### 2.1. Biological Function

Metals in biological systems are tightly controlled [27]. “Free” metal ions can lead to harmful side reactions, or can be scavenged by pathogens [28,29]. MTs are part of a complex network of proteins, metallochaperones, that are involved in the transport and storage of essential metals and maintenance of metal homeostasis [30,31]. MT has been shown to directly donate zinc to metallo-enzymes like carbonic anhydrase [32] and drive copper to cellular locales by a binding affinity gradient [33]. Knockout studies of mice *MT-1/2* genes produce mice unable to tolerate the influx of toxic metals that alter this homeostasis, although the deletion is not fatal in the absence of these toxic metals [34,35,36]. The deletion of these genes significantly decreases the ability to adapt to sub-optimal environmental metal concentrations [37,38,39].

Toxic metal sequestration is another proposed biological function of MTs. It is clear that MT confers a protective effect against cadmium intoxication [40,41]; MT was first isolated as a cadmium containing protein in an equine renal cortex [42,43]. In addition to cadmium, MT has been shown to bind arsenic under a wide variety of conditions [44,45,46,47,48] and to be associated with protection against arsenic related cancers [39,49,50,51], although isolation of As-MT species from in vivo studies remains elusive [52]. MT-3, the brain-specific isoform, has been shown to bind neurotoxic metals such as lead [53] and interact with proteins associated with neurodegenerative diseases [54,55,56]. In addition to homeostatic maintenance, it has been suggested that MTs having 20 free-SH thiols, contribute to redox signaling, and balance within the cellular environment [57,58,59]. RM-MS leverages the abundance of reactive thiols on MT to probe its solution structure without the need for high concentrations or crystallization. As MTs play a role both in the proliferation of tumors and resistance to chemotherapy, much interest has been given to analytical methods to detect and quantify MTs in biological samples [60,61].

### 2.2. Structural Characterization of MT in Various Metalation States

Our knowledge of the MT protein structure comes mainly from NMR studies, as this protein has been notoriously difficult to crystallize. The two X-ray crystal structures are of rat liver Cd_5_Zn_2_-MT-2 and yeast Cu_8_-MT, both fully metalated species of MT [62,63]. Much of the NMR data is of the fully metalated species of human and rat isoforms, especially with ^111,113^Cd [64,65,66]. Attempts to determine partially-metalated (Cd_2.9_MT-2) structures by NMR were only successful at lower pH, where we now know that cooperative cluster formation occurs [67], giving sharp distinct peaks compared to higher pH where a single broad undistinguished peak was observed [68]. Thus, NMR techniques are only useful when a stable metal-thiolate cluster has formed, even when only partially metalated [68] or supermetalated [69]. Under normal homeostatic conditions and at physiological pH, it is unlikely that MT would be able to form such stable clusters due to the constant shuttling of metal ions to higher affinity catalytic and structural sites on other metalloproteins. To obtain a better picture of the structure-function relationships of MT, more exotic techniques must be employed to probe the structure for the metal-free or partially metalated species.

### 2.3. Optical Techniques for MT Structural Determination: Circular Dichroism, UV-Visible Absorption, and Emission Spectroscopy

Optical techniques for investigating protein structure have been available for a long time and have been used extensively to characterize MT and its interactions with a variety of metals [70]. MT’s lack of aromatic amino acids limits the usefulness of far-UV absorption spectroscopy to investigate metalation status via the ligand-to-metal charge transfer band [71]. Even with drastic structural changes upon “supermetalation” with cadmium, the absorption spectra is relatively unchanged because the ligand-to-metal charge transfer (LMCT) chromophores are so similar that the superposition of the absorbance from the individual metal sites means that only an averaged spectrum is recorded [69]. Circular dichroism spectroscopy is more sensitive to structural changes, especially those that disrupt the symmetry of the metal-thiolate cluster and change the exciton coupling. This coupling results in a crossover point at 250 nm, the absorption maximum of the Cd-thiolate LMCT band. When the coupling is disrupted, meaning MT is either partially or super-metalated, the absorption maximum shifts to 250 nm and the crossover point to 240–245 nm as the symmetry dependent exciton coupling is lost. For emissive species like Ag, Au, or Cu-MTs, emission spectroscopy is useful in determining structure as solvent access to the thiolate clusters quenches the emission and provides information on the compactness of the protein conformation [72,73,74].

As we note above, these optical techniques are limited in their power for structure resolution for multi-metal binding MTs because they give an average response of all the species in solution. Also, these optical techniques are less useful for the spectroscopically silent zinc, perhaps the most biologically relevant metal that binds to MT and the one most commonly isolated for biological samples. When a distribution of multiple species is present, spectral features cannot be assigned to any one species. For example, when 2 mol eq. of Cd^2+^ are added to α-MT-1a, which can bind up to four divalent metals, apo- and Cd_1–4_α-MT are all present in varying amounts depending on pH conditions [67]. Mass spectrometry on the other hand can distinguish between each species that is present and measure their relative abundance which, when coupled with optical spectra, can accurately assign spectral features to specific MT species. In addition, techniques like ion-mobility mass spectrometry (IM-MS), tandem mass spectrometry (MS/MS), and residue modification mass spectrometry (RM-MS) can help to provide insight into the MT structure in apo-, holo-, and partially metalated states.

## 3. Mass Spectrometry for Structural Determination

Since the development of soft ionization techniques like electrospray and matrix assisted laser desorption/ionization (MALDI), the use of mass spectrometry in the field of protein structure and dynamics has grown exponentially. Unlike optical techniques, which examine global properties and observe an average response of all species present, MS techniques can simultaneously give information on species distribution and relative abundance, protein volume, surface area, and conformational changes via charge states [75], and information about specific regions within the protein and their solvent exposure via hydrogen/deuterium exchange (HDX) [76] or site specific covalent modification [77]. HDX is suited for larger proteins, with an extensive H-bonding network between backbone amide hydrogens which limits the rate of association/dissociation, resulting in a smaller mass change compared with disordered regions and those that are more solvent exposed. These techniques are reviewed in detail elsewhere [78].

## 4. Covalent Modification

A common criticism concerning the use of ESI-MS for the analysis of protein structure and conformation lies in the radically different surroundings the protein finds itself in when being analyzed compared to its native state. The native state, in solution, is buffered around neutral pH with an appropriate salt concentration, at approximately 37 °C and absent of chemical denaturants. The conditions of the electrospray process introduce high voltages, evaporating solvents causing a hyper-accumulation of salt ions and, finally, ionization of the protein itself and transition into a gas phase ion. The assumption that the conformations adopted in solution and under ESI conditions are the same may be a tenuous one.

An advantage of covalent modification is that it probes the conformation under solution conditions and changes thereafter during analysis and measurement of the gas phase ions have no effect on the modifications that occur prior to ionization. This is especially important for proteins whose structure is unstable and whose solution stabilized conformation may not reflect those conformers adopted during the electrospray process.

### 4.1. Covalent Modification to Identify Free Thiols

Cysteine is an important amino acid with high reactivity [79], able to coordinate a variety of soft metals [80], and form Cys–Cys covalent linkages that add stability to folded proteins [81]. Cysteine is also used to attach therapeutic and imaging moieties to proteins in vivo [82]. Covalent modification coupled with mass spectrometry can easily identify free cysteinyl thiols versus those that are oxidized to form crosslinks. New proteomic strategies also make use of specific covalent modifiers coupled with LC-MS and MS/MS to identify S-sulfenylation of cysteines and identify the most solvent exposed residues [83]. The redox chemistry inherent in quinones has been used for on-line tagging of free cysteines in ESI-MS analysis via a 1,4-Michael addition [84]. With the incorporation of a photo-active quinone, the protein backbone can be selectively fragmented with ultraviolet light for the analysis of solution structural properties or to monitor biological quinone post-translational modifications [85]. This technique can also be applied to other amino acids by leveraging thiol chemistry to selectively modify phosphorylated serine or threonine residues [86].

In the case of MTs, determining the number of free thiols under an assortment of different metal coordination numbers and geometries can help in structural determination. This is because the number of free thiols can be a good indicator of metal-cysteine cluster formation, as clusters involve bridging thiolates and, as a result, more metal ions can be coordinated with a smaller number of cysteine residues. Terminally coordinated metals require more cysteinyl thiols in MT and are considered to be more labile and thus available for donation to other metalloenzymes [67]. Also, when probing potentially new structures formed by the coordination of unusual metals and metalloids where the stoichiometry is not defined, the quantification of free thiols by cysteine modification and ESI-MS can establish defined Cys:Metal ratios [87].

Arsenical binding to metallothionein first reported by Le and coworkers [46] was characterized kinetically for free As(III) [45] and first reports indicated a stoichiometry of the fully saturated protein as an As_6_ species [46,88]. However, the coordination was only speculated upon, with As(III) unique in possessing a non-bonding electron pair, which likely forces the protein to adopt six tri-coordinate metal centers using 18 cysteinyl thiols and leaving two free-SH groups in human metallothionein. This was confirmed later by cysteine modification, and the As(SCys)_3_ structure formed for all intermediates (As_1–5_) in the metalation reaction as well, indicating the absence of bridging thiolates typical of metallothionein clusters [87].

**Figure 2:**
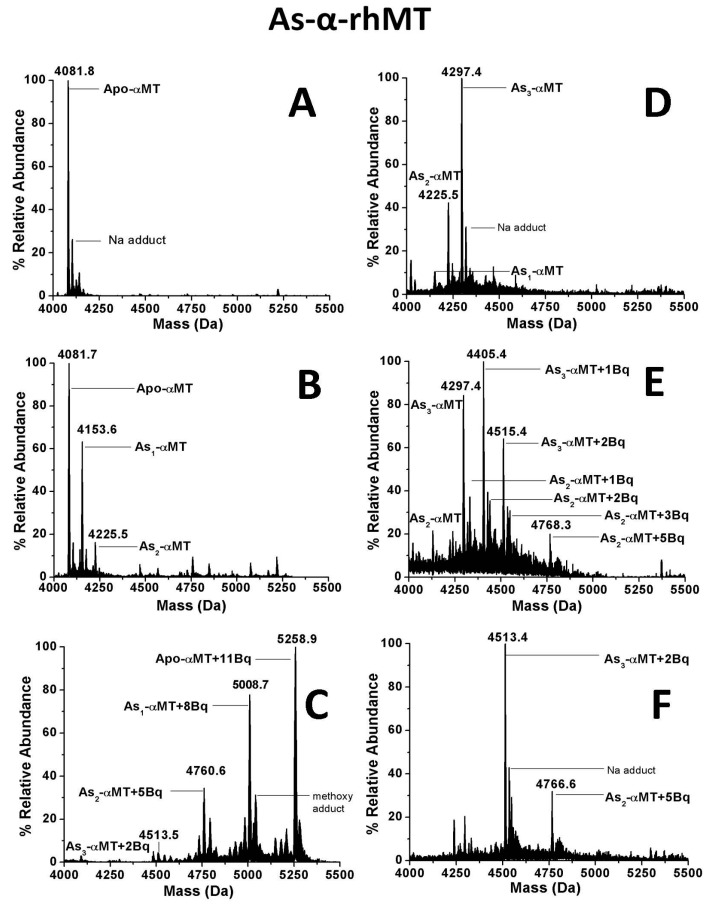
Deconvoluted electrospray ionization mass spectra (ESI-MS) of Arsenic-MT species (As_0-3_-α-MT-1a) covalently modified by *p*-benzoquinone (Bq). (**A**) Metal-free protein; (**B**) Partially metalated α-MT with As(III) with a large fraction of apo-protein; (**C**) Species in 1B fully modified by excess Bq; (**D**) Partially metalated α-MT with a larger fraction of saturated As_3_-α-MT; (**E**) Addition of 2 mol eq. of Bq to the species in 1D; (**F**) Species in 1D fully modified by excess Bq. Reproduced with permission from Irvine et al., 2013 [87].

During the non-cooperative metalation of metallothionein, a number of species are present simultaneously in solution with different numbers of bound metals, resulting in mass spectra with, usually, a normal distribution of peaks separated by the mass of the metal, as seen in Figure 2. Figure 2B,D show the distribution of As(III) bound to the α-domain of human metallothionein 1a at different points in the metalation reaction. Figure 2C shows the result of adding excess quinone, in this case *p*-benzoquinone (Bq), to the mixture where all free cysteine residues have been modified. Each peak (corresponding to the modified apo- and As_1–3_ species) is separated by the mass of three Bq molecules, indicating each As(III) is being coordinated by three cysteinyl thiols. When a different distribution of metalated species (Figure 2D) is modified, the result is the same (Figure 2F). This result indicates that the coordination of As(III) is fixed and does not adopt multiple geometries as in the case of Cu(I) binding to MTs. While Zn(II) and Cd(II) have fixed geometries, cluster formation via bridging ligands alters the ratio of bound vs. free thiols (i.e., Cd_4_(SCys)_16_ in terminal “beaded” structures vs. Cd_4_(SCys)_11_ in clustered structures) [89].

Figure 2 also highlights the power of ESI-MS for resolving multiple species present simultaneously in solution, even without the aid of chromatography as in GC-MS or LC-MS. Optical spectra may show global solution property changes due to the attachment of Bq to the protein but the number of species in Figure 2E would not be distinguished. A total of eight species, each with a unique mass due to differential metal loading and modification status, are easily resolved. In the data presented in Section 4.2, an even greater number of species is resolved in the ESI mass spectra.

The abundance of reactive thiols in apo-metallothioneins have been exploited in electrochemical studies [90], for functionalization and tethering of gold nanoparticles [91] and for their colorimetric detection [92]. The thiol-quinone reactivity has also been used to probe the structural properties of these hard to characterize proteins [93]. In addition to simple quantification of free thiols, covalent modification can be used to probe the structure and relative solvent exposure of specific residues in proteins that contain multiple cysteines.

### 4.2. Free Thiol Modification to Probe Domain Specificity in Metallothioneins

Domain specificity is a concept that has long been investigated in the case of metallothioneins with its two-domain structure consisting of a smaller, 9 cysteine β-domain and a larger 11-cysteine α-domain [94,95,96,97,98]. Domain specificity has been primarily investigated by NMR spectroscopy [95] and ESI-MS [98,99] due to the ability of both techniques to identify the domain in which the metal is bound. This has led to reports of certain isoforms and domains having specific metal binding properties, resulting in some isoforms and domains labeled as being metal-specific [97,100,101]. Conclusions reached from these in vitro studies were based on the metal binding constants and apparent metal preferences during titration. Attempts to study isoform and domain-specificity in vivo are hampered by the low concentrations, difficulty of extraction, and the ease of introducing and changing the metal distribution in the metallothionein during purification protocols. For example, over-expression of mammalian MT-3 in a recombinant system and supplementation of growth media with Cu(I) can lead to an apparent Cu-thionein character [97], although it is unclear how significant the MT-3 preference for Cu(I) would be in mammalian brain cells, with normal Cu(I) concentrations and with other competing Cu(I) binding proteins [54]. RNA expression of MT-3 was shown to be unaffected by Cu(I) supplementation in epithelial cancer cells, although cell type has a large influence on isoform expression [102]. Domain specificity presents a larger challenge, since RNA analysis cannot give information about intra-protein selection in response to metal stimuli. To address these questions, isolated domain competition experiments can be performed, although the elimination of the inter-domain linker raises questions about the strength of the chelate-effect of the entire protein and the barrier to cross domain metal exchange introduced by the separation of these domains in solution [98].

To determine the arrangement of metals in partially-metalated MTs without using isolated domain fragment experiments, the covalent modification of free thiols can be used in the full-length protein. In work by the Russell group, ESI-MS, covalent labelling with *N*-ethylmalemide (NEM), and IM-MS/MS was used to show MT-2a preferentially forms cadmium-thiolate clusters in the α-domain in both metalation and demetalation reactions [103]. Although some isoform-specific differences likely exist, MT-1a cooperatively forms cadmium clusters in the α-domain and this has been reported in both the full-length protein and the isolated α-domain fragment [67,89]. In competition experiments, a slight preference for α-domain metalation is observed that is exaggerated at lower pH [98]. Thus at least for the MT-1/2 isoforms cadmium metalation is α-domain specific, a phenomenon that becomes more apparent at lower pH.

**Figure 3:**
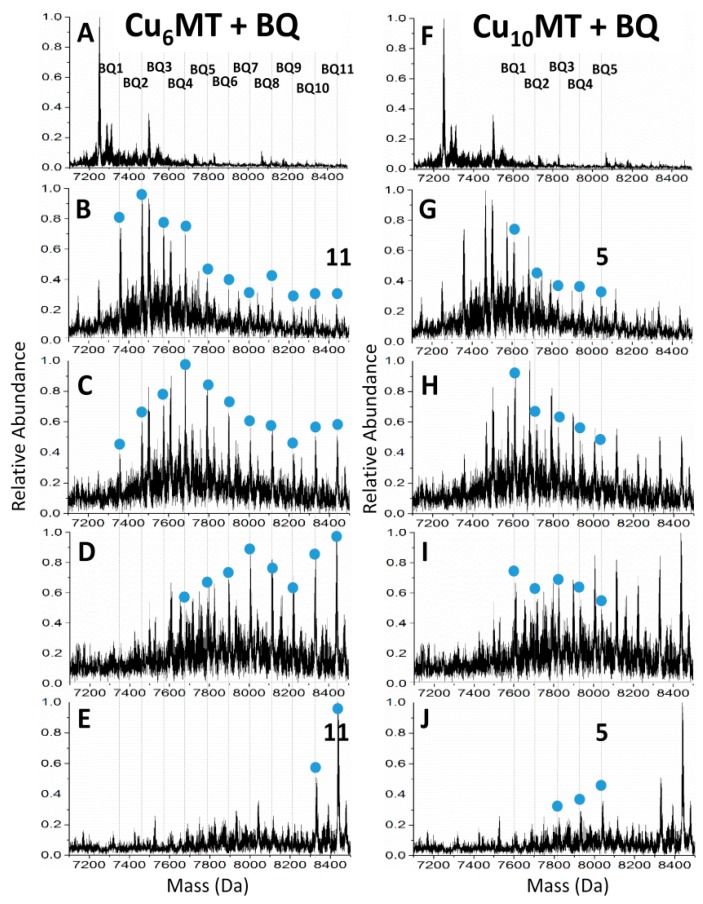
Free cysteine modification with *p*-benzoquinone of a Cu-MT solution containing mainly Cu_6_ and Cu_10_-MT species. Both columns show the same spectra with different peaks labeled and highlighted with blue dots. Bq modified species for Cu_6_-MT are shown in (**A**–**E**) and the modified species for Cu_10_-MT are shown in (**F**–**J**). Reproduced from ref. [108] with permission from the Royal Society of Chemistry.

In addition to cadmium and arsenic metalation, covalent modification was used to probe the structures adopted during Cu(I) metalation of the MT-1a isoform. The multiple geometries that Cu(I) can adopt in MT with 20 available Cys-ligands makes structural characterization extremely difficult. Like the other MTs, the only crystal structure reported to date is that of the metal-saturated form and, for Cu(I) MTs, only for yeast-MT which is saturated as Cu_8_-MT, whereas, the mammalian isoforms can accommodate up to 20 Cu(I) ions. Recently, the metalation and folding pathway of Cu(I) metalation of apo-MT1a was probed using emission and CD spectroscopy, and ESI-MS coupled with covalent modification by *p*-benzoquinone (Bq). Through emission and CD spectroscopy, the overall conformational changes were monitored and ESI-MS helped to identify the metal coordination number and distribution of species that gave rise to the optical spectra. These data allowed identification of species that correspond to known cluster structures and the modification of free thiols proved that the Cu_6_ cluster was formed in the β-domain and the Cu_10_ species consists of the Cu_6_ cluster in the β-domain and a Cu_4_ cluster in the α-domain (Figure 3). This domain specificity showed different pH dependence than that of cadmium, where cluster formation was most dominant between pH 5.3 and 7.4 and followed a more non-cooperative, terminally bound pathway above and below this range.

RM-MS is not particularly suitable for probing differences between MT isoforms, as long as those isoforms have identical numbers of cysteine residues and bind metals with the same Cys-metal stoichiometry. These major differences are unlikely between structurally similar isoforms from mammalian species, the major inter-isoform difference is considered to be with metal preference and spatial distribution biological context [104,105]. The mammalian MT family is considerably less diverse than amongst other biological strata [106]. Interestingly, ionization efficiencies vary widely between the similar mammalian MT isoforms, and even between the two domains of MT1a which require external concentration verification in competition and affinity experiments [32,98,107].

### 4.3. Covalent Modification to Probe Solution Structure and Protein–Protein Interactions

Modification of reactive residues has proven in the past to be a useful albeit mostly low-resolution tool to probe the structures of hard to characterize proteins, this method being another tool in the arsenal of techniques that are needed to piece together an understanding of how ill-defined proteins behave. One strategy is to use cross-linking reagents to tether two interacting proteins together. Once connected by the linker, the dimer can be proteolytically digested or fragmented via tandem MS and analyzed to identify the residues which participated in the cross-linking and thus were likely involved in the protein-protein interaction [109].

**Figure 4:**
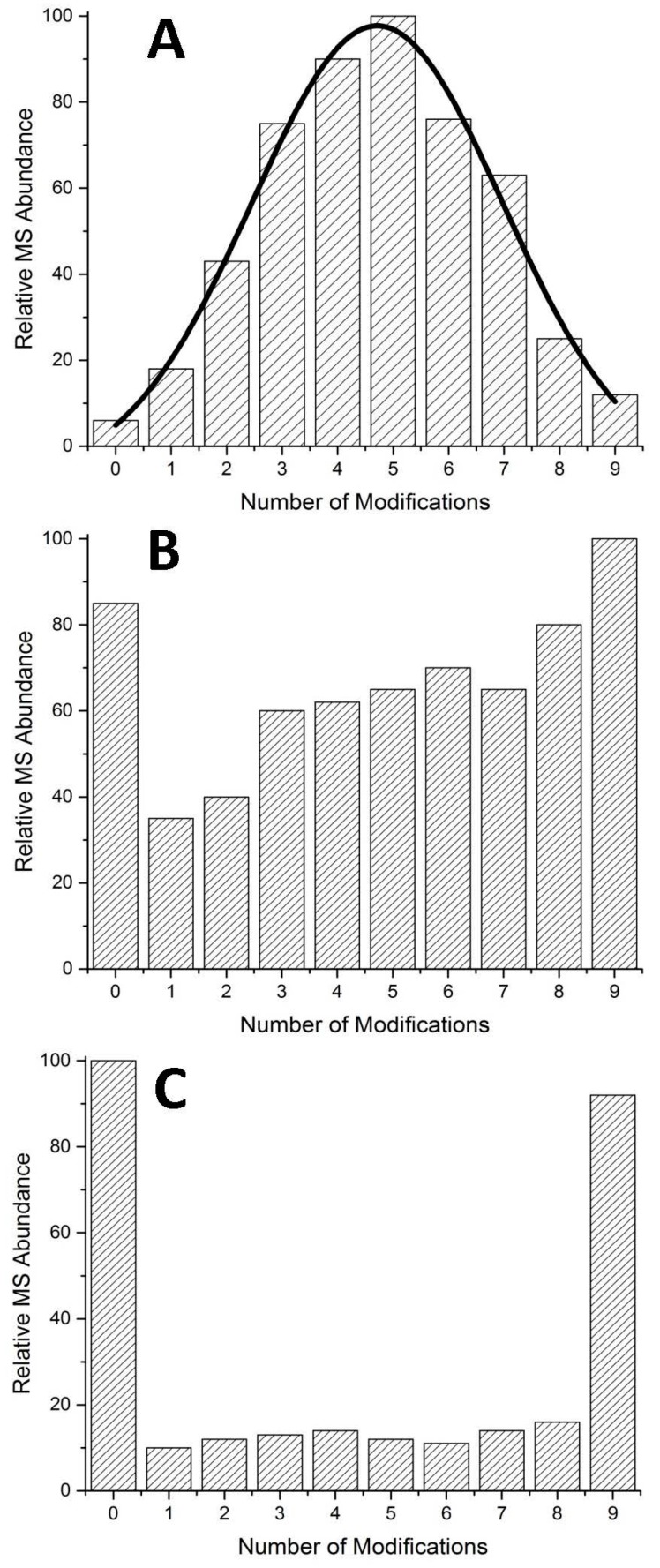
Examples of possible modification distributions in typical mass spectra. (**A**) Normal distribution of modified species where small amounts of apo- (0 modifications) and fully modified species (9 modifications) exist simultaneously; (**B**) semi-cooperative pattern, has significant amounts of the apo-species, intermediates (1–8 modifications), and fully modified species; (**C**) cooperative pattern, where few intermediates exist and the spectra is dominated by the apo- and fully modified species.

In addition to chemical cross-links, analysis of modification patterns can be helpful in identifying protein–protein interactions in peptides where many residues can be modified, like MTs. In cases where all modifiable residues (in the case of MTs, cysteine) are equally accessible by the modifying agent, the result will be a normal distribution of modified species (Figure 4A). Where unequal access exists, an altered pattern of modification will exist. This is exemplified in Figure 4B,C. At low pH most proteins denature and this can be easily monitored by optical methods with the loss of secondary structure or via ESI-MS in the appearance of higher charge states. Because of MT's small size, the shift in charge states is minor and due to its lack of secondary structural features in the absence of metals, no change in the optical spectra is observed. The modification profile however follows a stochastic distribution, indicating equal access to all cysteine residues. Arsenic metalation of MTs is unique in that it metalates even at low pH, albeit at a slow rate able to be monitored by ESI-MS [45].

In the presence of As(III) ions, even at low pH, the modification profile of the partially-metalated species shifts drastically to one that can be described as semi-cooperative [44]. That is, the unmodified species exists in solution in roughly equal amounts as all partially modified species, up to species where all free cysteine residues not involved in As(III) coordination are covalently bound by Bq. This is an indication that even under denaturing conditions, the introduction of a coordinated metal ion induces the folding of MT that creates unequal solvent access to some free cysteine residues. This result is not unexpected as it is well known that the introduction of metals creates a more rigid structure even if only partially-metalated [71,110]. What is surprising about the data is that the modified apo-MT species follow the same pattern.

**Figure 5:**
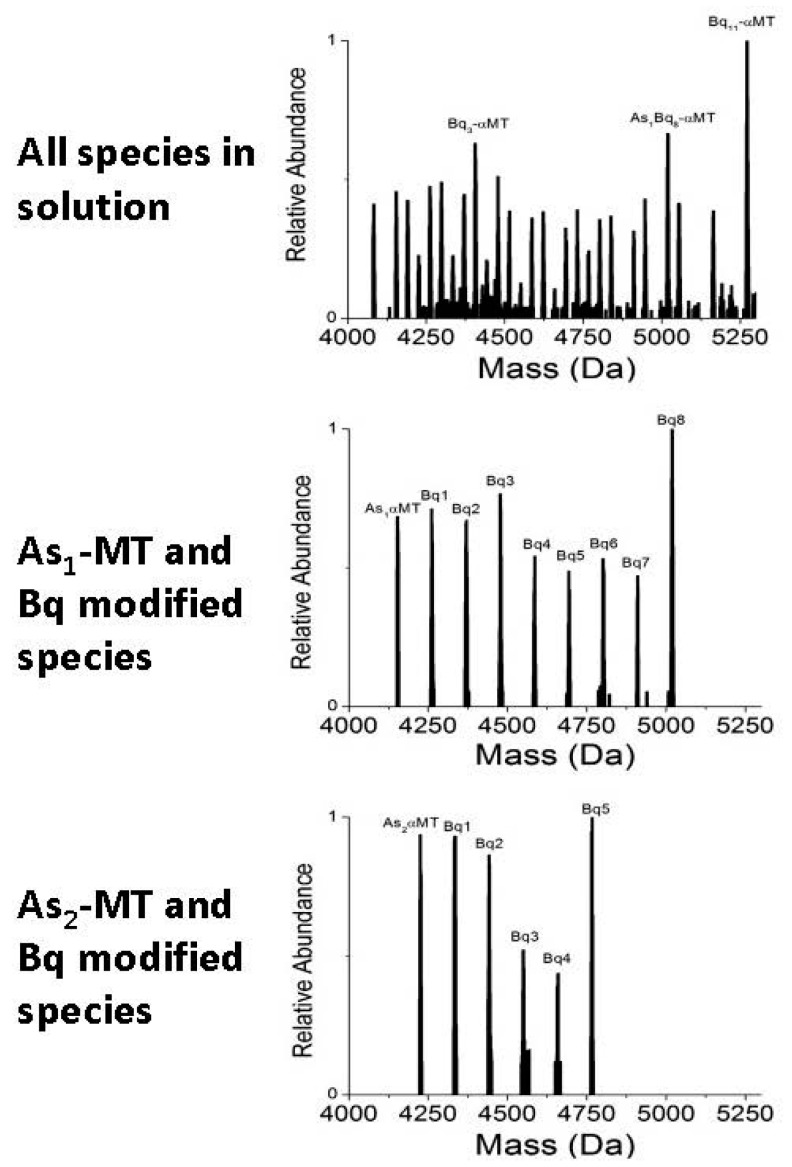

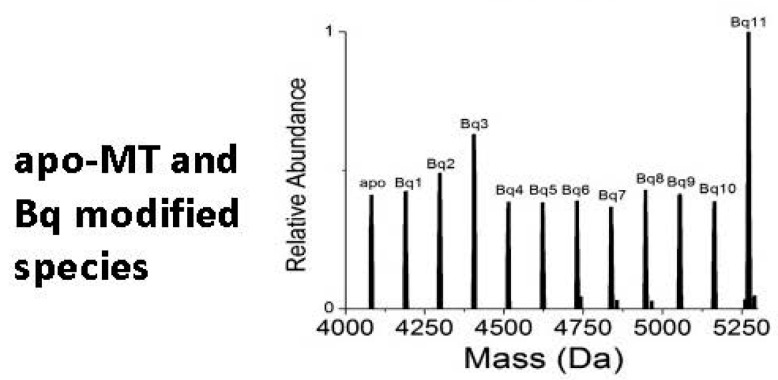
Modification of apo-As_1_ and As_3_-αMT solution with *p*-benzoquinone (Bq). The original spectra after modification is shown at the top. The individual species and their modifications are isolated from the top spectra and are shown below in the left column. Adapted with permission from Irvine et al., 2013 [44].

The semi-cooperative pattern of modifications of the apo-MT species is highlighted in Figure 5. Again, this indicates that the cysteine residues in apo-MT, where no metal is coordinated, are differentially shielded by some means. Since the reaction was carried out, and mass spectra recorded, under denaturing conditions, it is not the native folding that is causing the unequal access and resultant modification profile. The exchange of As(III) ions between proteins in solution is a slow process and the residual structure left after As(III) has left is likely to be very short-lived under denaturing conditions. A more plausible explanation is that protein-protein interactions facilitated by As(III) coordination causes certain cysteine residues to be shielded from the bulk solution and therefore are unavailable for modification.

The results from the cysteine modification experiments highlight the importance of metal coordination to MT folding, even under extreme conditions. Previous experiments showed that As(III) transfer between MT proteins must proceed via a direct transfer rather than a dissociative/associative mechanism [47]. The cysteine modification experiments was able to probe the interactions in solution and was easily measured via ESI-MS. While the results are at times difficult to interpret, a simplified way to examine the data is to always compare the modification to a normal distribution of modified species. Any deviation indicates the differential shielding of cysteines within the protein with larger deviations pointing towards large discrepancies between individual cysteine residues in terms of solvent accessibility.

## 5. Probing Protein Conformation via Differential Modification

The conformation adopted by MT, one that is fluxional and ill-defined, has been shown to be essential for metalation kinetics [111]. This is somewhat counter-intuitive, since a more open conformer where the cysteine residues are most exposed to the solvent and incoming metal ions might be expected to metalate faster. However, key to the metalation mechanism of MTs, especially when binding cadmium, is the formation of cadmium-thiolate clusters which involve both bridging and terminal cysteine residues. The more compact conformer(s) found under native conditions, while they may not represent all conformers found under these conditions [110], likely orient the cysteine residues in a way that is “primed” for metal binding and cluster formation. That is, the cysteine residues are aligned so that the bridging cysteines are in, or close to, the optimal position to form a bridging bond between two cadmium ions. Once formed, the cadmium-thiolate cluster is known for its stability and amenability to determination by ^113^Cd NMR and other methods.

To better understand the origin of these differential modification patterns, the reaction can be modeled using a series of sequential, bi-molecular reactions which can give simulated mass spectral data that include species abundances at different points in the reaction. When the modeled Ks of the reaction reproduce the experimental mass spectra to a high degree of similarity, the relative values of the Ks can be compared and conclusions can be drawn. In the case of the differential modification of α-MT-1a by Bq, the Ks revealed a series of declining values under denaturing conditions which is expected as the loss of binding sites as the reaction proceeds results in the statistical decrease of binding constants. Under native conditions, the relative constants do not decrease but increase slightly. This can be explained by the opening or denaturation of the compact conformers by the modification of cysteinyl thiols by the bulky Bq molecule. There are two major factors in addition to the intrinsic chemical nature of the reaction that govern the rate at which cysteine residues are modified:

(1) The statistical availability of free thiols. During the early stages of the modification of a peptide, the abundance of free thiols makes a reaction more likely since there are more “binding sites” for the modification reagents. Since there are more places on the protein that are capable of reaction, a successful collision between a free cysteine and an alkylation reagent is more likely. As the cysteine residues are modified, the likelihood of a successful collision decreases due to the increasingly rare potential reaction sites on the protein.
(2) The steric accessibility of free thiols. It is well-known that the semi-hydrophobic nature of cysteine allows for marked differences in solvent accessibility depending on the folded state of the protein [112,113,114,115]. The more solvent that has access to these residues, the faster the reaction rate [112]. During the modification reaction of MTs, the compact conformer extends, exposing previously buried residues and increasing their solvent accessibility and therefore, the rate of modification. This is likely the origin of the semi-cooperative nature of the modification reaction of MTs under native conditions.

Questions then arise about the nature of the modifier and its effect on protein conformation and reaction profile.

In order to test differences in cysteine modification profiles, three common covalent cysteine modifiers were tested: *p*-benzoquinone (Bq), *N*-ethylmalemide (NEM), and iodoacetamide (IAM). These three modifiers have different sizes, hydrophobicities, and reaction mechanisms that are well characterized [116,117,118].

**Figure 6:**
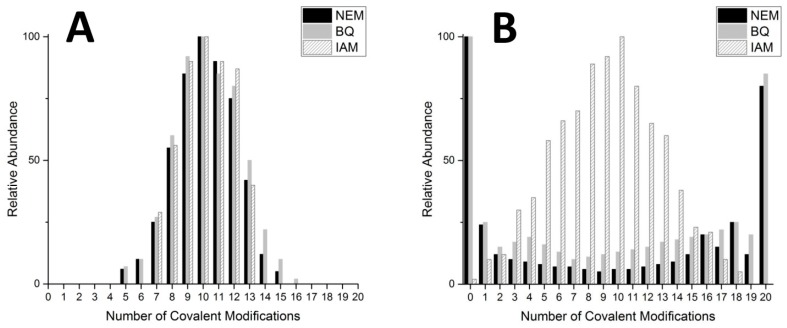
Modification profiles of *N*-ethylmalemide (NEM), *p*-benzoquinone (Bq), and iodoacetamide (IAM) cysteine alkylation reagents with βα-MT-1a under denaturing (**A**) and native (**B**) conditions. Relative abundance taken from deconvoluted ESI-MS data after the reaction of 10 mol eq. of each of the modifiers. Adapted with permission from Irvine et al., 2017 [119].

**Figure 7:**
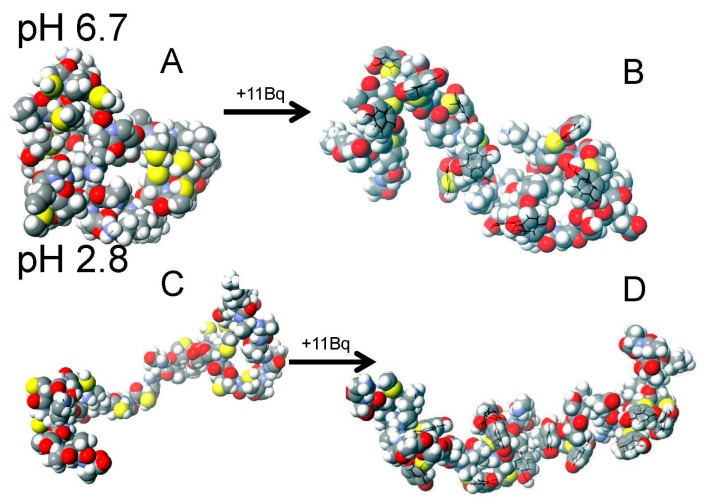
Molecular mechanics, molecular dynamics (MM/MD) simulation of energy minimized α-MT conformations under native (**A**,**B**) and denaturing (**C**,**D**) conditions. Apo-αMT is shown in **A** and **C** and fully modified Bq_11_-α-MT in **B** and **D**. Adapted with permission from Irvine et al., 2015 [111].

The two larger modifiers, NEM and Bq, resulted in very similar modification profiles (Figure 6). The origin of these cooperative-like patterns lies in the disruption of the native globular, random coil configuration by the large and more hydrophobic cysteine modifiers. The disruption of the compact confomer causes the remaining free cysteines to become more exposed to the solvent and more readily react with more incoming NEM or Bq molecules. Visual representations of these two conformers are presented in Figure 7 with the compact, globular conformation in Figure 7A and the extended conformation in Figure 7C.

Figure 7 shows the large change in conformation upon Bq modification under native conditions and the minor change under denaturing conditions. Under denaturing conditions, the protein already exists in an extended conformation and the modification of cysteine residues does little to change this, in contrast to the compact conformation where modification opens up the structure.

## 6. Kinetic Data from ESI-MS and Cysteine Modification

The data discussed in the previous section are essentially “snapshots” of the reaction; the progress reports that would be observed if excess modifier were added and ultra-high speed spectra recorded. Instead we used a step-wise addition where we run out of modifier and the distribution of modified species is recorded at each point. The modification status is governed by the relative kinetics of the reaction to modify each cysteine. In a paper by Irvine et al. [111], relative rate constants for each of the 11 modification reactions of the α-fragments of MT were calculated and the trend in relative ks was used to interpret the spectra.

In addition to reporting the relative kinetics of the intrinsic cysteine alkylation reaction, this reaction can be modified to probe demetalation through the competition of metals with modifying reagents. By mixing fully metalated Cd_7_-MT2a with excess NEM, Chen et al. were able to observe major Cd_x_NEM_y_ species form as the ratio of NEM:MT was increased [120]. Through monitoring the progress of this slower displacement reaction via ESI-MS and subsequent analysis though collision induced dissociation, ion mobility mass spectrometry (MS-CID-IM-MS), they were able to determine the kinetic rate constants of each of the seven displacement reactions and identify the most labile cysteines in MT2a [120]. This approach is novel in its site specificity for probing cysteine accessibility and involvement in metal binding and can help to determine metal binding mechanisms and structural conformations in exquisite detail.

## 7. Conclusions

Structural information regarding MTs has been hard to come by due to its highly dynamic nature in the absence of metal clusters. Recently, advances have been in residue modification mass spectrometry in order to probe the hard-to-characterize structure of apo- and partially metalated MTs. By using different site and cysteine specific alkylation reagents, time dependent ESI-MS, and step-wise “snapshot” ESI-MS, we are beginning to understand the dynamics of the conformers of apo-MT and related species. Recent studies have also highlighted the importance of the apo-MT structure for metalation kinetics. Coupled with other mass spectrometric techniques like IM-MS, the structure of apo-MT once thought to be non-existent is beginning to become clear. We expect increasing resolution in our picture of the structural conformations of metal-free MTs as this technique is more widely adopted and combined with other promising tools for structural elucidation. In addition, these methods could be extended to other intrinsically disordered proteins with significant amounts of reactive residues, adding another tool to the toolbox available for structural investigations.
